# Functional Neuroanatomy of Second Language Sentence Comprehension: An fMRI Study of Late Learners of American Sign Language

**DOI:** 10.3389/fpsyg.2018.01626

**Published:** 2018-09-06

**Authors:** Lisa Johnson, Megan C. Fitzhugh, Yuji Yi, Soren Mickelsen, Leslie C. Baxter, Pamela Howard, Corianne Rogalsky

**Affiliations:** ^1^Department of Speech and Hearing Science, Arizona State University, Tempe, AZ, United States; ^2^Interdisciplinary Graduate Neuroscience Program, Arizona State University, Tempe, AZ, United States; ^3^Barrow Neurological Institute and St. Joseph's Hospital and Medical Center, Phoenix, AZ, United States

**Keywords:** sentence comprehension, sign language, fMRI, bilingual, language

## Abstract

The neurobiology of sentence comprehension is well-studied but the properties and characteristics of sentence processing networks remain unclear and highly debated. Sign languages (i.e., visual-manual languages), like spoken languages, have complex grammatical structures and thus can provide valuable insights into the specificity and function of brain regions supporting sentence comprehension. The present study aims to characterize how these well-studied spoken language networks can adapt in adults to be responsive to sign language sentences, which contain combinatorial semantic and syntactic visual-spatial linguistic information. Twenty native English-speaking undergraduates who had completed introductory American Sign Language (ASL) courses viewed videos of the following conditions during fMRI acquisition: signed sentences, signed word lists, English sentences and English word lists. Overall our results indicate that native language (L1) sentence processing resources are responsive to ASL sentence structures in late L2 learners, but that certain L1 sentence processing regions respond differently to L2 ASL sentences, likely due to the nature of their contribution to language comprehension. For example, L1 sentence regions in Broca's area were significantly more responsive to L2 than L1 sentences, supporting the hypothesis that Broca's area contributes to sentence comprehension as a cognitive resource when increased processing is required. Anterior temporal L1 sentence regions were sensitive to L2 ASL sentence structure, but demonstrated no significant differences in activation to L1 than L2, suggesting its contribution to sentence processing is modality-independent. Posterior superior temporal L1 sentence regions also responded to ASL sentence structure but were more activated by English than ASL sentences. An exploratory analysis of the neural correlates of L2 ASL proficiency indicates that ASL proficiency is positively correlated with increased activations in response to ASL sentences in L1 sentence processing regions. Overall these results suggest that well-established fronto-temporal spoken language networks involved in sentence processing exhibit functional plasticity with late L2 ASL exposure, and thus are adaptable to syntactic structures widely different than those in an individual's native language. Our findings also provide valuable insights into the unique contributions of the inferior frontal and superior temporal regions that are frequently implicated in sentence comprehension but whose exact roles remain highly debated.

## Introduction

The neurobiology of sentence comprehension has been extensively studied for decades. Yet, there remains intense debate regarding the nature and specificity of contributions to sentence comprehension of several left fronto-temporal brain regions. The vast majority of the previous work regarding the neural correlates of sentence comprehension has investigated spoken languages. This work has identified a left-lateralized fronto-temporo-parietal network that is activated by the presence of sentence structures, compared to a variety of acoustic controls, with the most common regions of interest in Broca's area (posterior 2/3 of the left inferior frontal gyrus), the posterior superior temporal gyrus, and anterior temporal cortex (Dronkers et al., 2004; Hickok and Poeppel, 2007; Magnusdottir et al., 2013). Although these regions are frequently identified in studies of sentence comprehension, their respective contributions remain controversial. For example, the role of Broca's area has been attributed to cognitive resources including working memory and cognitive control (Just et al., 1996; Kaan and Swaab, 2002; Novick et al., 2005; Rogalsky et al., 2008; Pettigrew and Hillis, 2014), hierarchical structure-building (Friederici, 2009; Makuuchi et al., 2012) and syntax-specific resources (Grodzinsky, 2000; Grodzinsky and Santi, 2008). Anterior temporal contributions have been attributed to combinatorial semantics (Dapretto and Bookheimer, 1999; Vandenberghe et al., 2002; Ferstl et al., 2005; Pallier et al., 2011; Bemis and Pylkkänen, 2013), semantic processing more generally (Wong and Gallate, 2012; Wilson et al., 2014), prosody (Phillips et al., 1998; Adolphs et al., 2002; Friederici et al., 2003; Humphries et al., 2005; Johnstone et al., 2006), and basic syntactic processing (Humphries et al., 2006; Rogalsky et al., 2011; Herrmann et al., 2012). Posterior superior temporal regions also have been implicated by many of these same studies in combinatorial semantics, syntax and prosody (Humphries et al., 2006; Griffiths et al., 2013; Wilson et al., 2014), as well as in lexical and phonological processing (Damasio et al., 2004; Hickok and Poeppel, 2007; Graves et al., 2008).

The present study aims to investigate the response properties of these spoken language sentence-processing regions to American Sign Language (ASL) sentences in normal hearing adults who are novice ASL learners. Previous studies of the neural substrates of sign languages (i.e., visual-manual languages) have provided valuable insights into the specificity and function of language processing brain networks. Sign languages, like spoken languages, have complex grammatical structures, sublexical features, and many other similar linguistic properties (Emmorey et al., 2002; Sandler and Lillo-Martin, 2006). Thus, by comparing across languages in different modalities, major strides have been made regarding understanding the overall organization and properties of the human brain's language systems independent of modality (Emmorey and McCullough, 2009). Studies investigating the neural substrates of sign languages in native deaf signers (i.e., individuals who have been deaf from a young age and learned a sign language from birth or in childhood) have found that native signers and native speakers engage highly overlapping brain networks during both language production and comprehension. For example, functional MRI studies of native deaf signers consistently indicate that ASL sentences activate the classic left hemisphere language network, including Broca's area, and anterior and posterior portions of the left superior temporal gyrus (Neville et al., 1997; Corina and McBurney, 2001; Emmorey et al., 2003; Sakai et al., 2005). In addition, studies of native deaf signers who have focal brain damage due to a stroke indicate that sign aphasias result from lesion patterns very similar to those typically associated with spoken language aphasias (e.g., left frontal damage in Broca's aphasia, left temporal/parietal damage in fluent aphasias, etc.) (Hickok et al., 1998a; Emmorey et al., 2007; Rogalsky et al., 2013).

Previous studies of bimodal bilinguals who acquired both sign and spoken languages early (i.e., before puberty) also indicate substantial overlap between brain regions engaged in comprehending spoken and signed words (Petitto et al., 2000; MacSweeney et al., 2002, 2006, 2008; Leonard et al., 2013). For example, Mayberry et al. (2011) find the same left-lateralized network engaged in lexical-semantic processing for both early acquired spoken and sign languages. Event related potential (ERP) findings also indicate that grammatical and semantic errors elicit similar ERP responses for both sign and spoken languages in native hearing signers (Neville et al., 1997; Bavelier et al., 1998). Together, these sign language findings suggest that when acquired early, spoken and sign languages share abstract linguistic properties and that the components of language processing and acquisition occur largely independent from modality of input (Hickok et al., 1998a; Corina and McBurney, 2001; Emmorey, 2002), although there are some known language modality differences, particularly in parietal regions (Emmorey et al., 2007, 2014; Pa et al., 2008).

Studying late bimodal bilingualism (e.g., a native English-speaking adult learning American Sign Language) can lead to a better understanding of the neurobiology of late second language acquisition (Leonard et al., 2013). For example, spoken language bilingual studies cannot alone determine if how the adult brain adapts to novel lexical-semantic mappings and syntactic structures is dependent upon the modality of presentation, or if, for example, auditory speech regions involved in lexical processing can also adapt to lexical-semantic processing of manual signs in adulthood (i.e., well after any language critical period). No previous studies to our knowledge have investigated syntactic or sentence-level processing in late bimodal bilinguals, but the few existing neuroimaging studies addressing lexical-semantic processing in late bimodal bilingualism are summarized below.

Leonard et al. (2013) examined the neural correlates of late L2 ASL in hearing L1 English speakers who completed 40 h of ASL college-level coursework. Leonard et al. found that a single-word semantic task (word-picture matching) in spoken English, written English, and ASL, all evoked a very similar left-lateralized fronto-temporal network, and that ASL also engaged a right inferior parietal region more so than spoken or written English. Right inferior parietal regions also previously have been identified as an ASL-specific area of activation in early ASL-English bilinguals (Newman et al., 2002). Seemingly in conflict with these findings of right hemisphere engagement during late L2 ASL acquisition, Williams et al.'s. (2016) longitudinal study of word-level processes in adult novice L2 sign language learners found that as a semester of ASL instruction progressed, left hemisphere activation increased while right hemisphere involvement decreased, in part leading to an increase in overlap with L1 neural correlates. This right-to-left hemisphere shift in activation as a function of proficiency is also seen in L2 spoken languages (Dehaene et al., 1997; Meschyan and Hernandez, 2006), and may reflect reduced engagement of domain-general cognitive control and attention resources. For example, lower L2 sign proficiency was associated with bilateral activation in the caudate nucleus and anterior cingulate cortex, neural structures previous implicated in spoken language control and cognitive control more generally (Friederici, 2006; Abutalebi, 2008; Zou et al., 2012). Thus, the initial right parietal involvement but overall decline in right hemisphere involvement may reflect a shift from more domain-general resources to L1 processing networks for ASL L2, in addition to engagement of an “ASL-specific” right parietal region.

Overall the findings from late L2 ASL studies of word-level processing suggest that lexical-semantic networks are amodal and can quickly adapt to lexical-semantic information coming from a novel modality, particularly in individuals with higher L2 sign proficiency. However, it remains unknown if this finding expands to sentence structure: can the brain networks that support sentence-level syntactic processing in spoken languages also adapt to the visual-spatial syntactic cues of a signed language?

One might assume that if lexical resources can adapt to a different modality, then syntactic resources logically could do so in kind. However, spoken language syntactic cues include temporal and/or auditory-verbal information such as word order, conjugation and declension (morphosyntactic cues), punctuation and/or prosodic inflections, while sign language syntactic cues come in the form of visual-spatial information including location in space, movements, and face, head, and body positions. There is a robust literature of findings indicating distinct neural resources that process visual-spatial information compared to verbal information, the most general of which is that the right hemisphere is more tuned to visual-spatial information while the left hemisphere is more specialized for language (Gazzaniga, 1989, 2000). For example, dissociations of visual-spatial and linguistic (including syntactic) impairments after brain injury or disease are well-documented in spoken language users (e.g., Glosser and Goodglass, 1990; Mosidze et al., 1994; Baldo et al., 2001)^1^. Evidence from spoken language bilingual studies also suggests a possible dissociation between the adaptability of semantic and syntactic neural resources: age of L2 acquisition has a greater effect than proficiency on the overlap of the neural correlates of L1 and L2 syntactic processing, while semantic neural resources are more affected by proficiency than age of acquisition (Weber-Fox and Neville, 1996; Wartenburger et al., 2003). Thus, it is unknown if the visual-spatial nature of syntactic information in sign languages affects the ability of established spoken language syntactic processing resources to adapt to new syntactic cues. Further, it is unclear if understanding the flexibility of spoken L1 sentence-processing resources in ASL L2 is a worthy pursuit to better understand the response properties and adaptability of these syntactic resources critical for human language.

One confound in investigating the neural differences and similarities of a spoken L1 and a late sign L2 is that it may not be clear if effects are due to differences in proficiency, age of acquisition, and/or the languages being in different modalities and thus having different syntactic features. However, there is a large literature in spoken language bilingualism on the variables of proficiency, age of acquisition, and syntactic similarity to inform findings in bimodal bilinguals (for thorough reviews, please see Caffarra et al., 2015 and Kotz, 2009). Some of the most relevant findings in spoken language studies for interpreting our findings in this present study of L2 ASL syntactic processing include: (1) there is a “syntactic similarity effect” in that the similarity of the neural correlates of L1 and L2 are greater for languages that have similar syntactic features and structure types (Ojima et al., 2005; Chen et al., 2007; Jeong et al., 2007), (2) late L2 learners can exhibit native-like ERP signatures during L2 comprehension when proficiency is high (Rossi et al., 2006; Tanner et al., 2013), (3) significant bilateral superior temporal activation is found for most L1 and L2s regardless of proficiency or age of onset although activation in this region is positively correlated with proficiency (Perani et al., 1998; Jeong et al., 2007), and (4) the inferior frontal gyrus is engaged more in late L2 than in L1 comprehension, particularly for low L2 proficiency (Rüschemeyer et al., 2005, 2006; Hernandez et al., 2007; Jeong et al., 2007). Together the last two points suggest what Rüschemeyer et al. (2005, 2006) call a “trade off” between inferior frontal and superior temporal involvement, with greater IFG involvement as a function of L2 and lower proficiency and greater STG involvement for L1 and higher proficiency L2. These findings likely point to the IFG supporting the learning of syntactic rules (as IFG also has been identified in studies of artificial grammar, e.g., Opitz and Friederici, 2004; Friederici et al., 2006).

The present study compares the brain networks engaged in ASL sentence comprehension in adult novice L2 ASL learners to those involved in L1 spoken language sentence comprehension. This work expands the very small literature on the neurobiology of late bimodal second language acquisition; to our knowledge no previous study has examined the brain networks supporting ASL sentence comprehension in late bimodal bilinguals. Our aim is to characterize the functional plasticity of auditory sentence processing regions for sentence structures that have similar grammatical complexity, but are represented visually and visuo-spatially, in hearing adults who are novice sign language users. We hypothesize the following: (1) ASL and English sentences will activate highly overlapping frontal-temporal networks, (2) ASL sentences will elicit more activation in Broca's area than English sentences, likely because of Broca's area contributing to sentence comprehension as a cognitive resource, (3) ASL and English sentences will engage sentence processing resources in anterior and posterior temporal regions to a similar degree, and (4) ASL sentence comprehension proficiency will be negatively correlated with right hemisphere involvement.

## Methods

### Participants

Twenty participants were recruited from Arizona State University's (ASU) American Sign Language undergraduate program in the Department of Speech and Hearing Science. Participants (one male; age range = 18–31 years, mean age = 21.4 years) met the following criteria: native speaker of American English, mono-lingual, right handed, previous completion of two semester-long (15 week) introductory courses of American Sign Language (i.e., ASL 101 and 102 at ASU or equivalent at a community college) and current enrollment in an upper division ASL course at the time of participation (ASL 201: *n* = 8, ASL 202: *n* = 12). The large percentage of participants who were female reflect the gender ratio in the ASL courses sampled. Participants reported no history of neurological or psychological disease, which was corroborated by a clinical neuroradiologist who reviewed the structural MRI scans. This study was carried out in accordance with the recommendations of the Institutional Review Boards of ASU and St. Joseph's Hospital and Medical Center with written informed consent from all subjects. All subjects gave written informed consent in accordance with the Declaration of Helsinki. The protocol was approved by the ASU Institutional Review Board and the St. Joseph's Hospital and Medical Center Institutional Review Board.

### Stimuli

During fMRI acquisition, blocks of four types of stimuli were presented: ASL sentences, ASL word lists, English sentences, and English word lists. Each are described below. All ASL stimuli were digitally recorded and edited using AdobeSuite Premiere® software.

#### ASL sentences

The words used to generate the ASL sentences were taken from common nouns, verbs, adjectives, and adverbs presented in vocabulary lists used by first year ASL courses in which participants were previously enrolled (i.e., ASL 101, ASL 102). From this vocabulary corpus, 15 ASL sentences were generated by a fluent ASL instructor (author P.H.) who added place, movement, and expression (i.e., the inflectional morphology). P.H. has been a college ASL instructor for over 30 years, and is a certified interpreter. The sentences also were reviewed by a native deaf signer (age 22, acquired ASL from birth, not a student or colleague of P.H.).

ASL sentences ranged from 5 to 10 signs long (*M* = 7.6) and consisted of a variety of sentence structures and clause types (e.g., “Grandmother was upset that father forgot to pick up his daughter from school,” “Yesterday the secretary answered the phone when the boss left”). A total of 15 sentences were used during scanning, while a different 10 sentences were used for the ASL proficiency measure, described below. During scanning each ASL sentence presentation block consisted of one ASL sentence and the mean duration of each block was 6.9 s (5–8 s).

#### ASL word lists

Fifteen ASL word lists were generated from the common nouns utilized in the ASL sentences. Each word list was comprised of seven nouns. All ASL sentences and word lists were signed by the same fluent signer (P.H.). During the word lists, the signer returned her hands to her lap between each signed word. The mean duration of each ASL word list block was 14.5 s (14–16 s).

#### English sentences

Thirty English sentences were generated by translating into English the 15 ASL sentences used in ASL sentence blocks, plus 15 additional sentences generated from the same ASL vocabulary corpus. English sentences ranged from 8 to 16 words (*M* = 11.3). Presentation of the English stimuli are based on Fedorenko et al. (2011) language localizer paradigm: English stimuli were presented as white text against a dark gray background. Each word in the sentence was visually presented for 350 ms. Sentences ranged in duration from 2.8 to 5.6 s (*M* = 3.9 s). Each English sentence presentation block consisted of two English sentences, which were separated by a 700 ms fixation cross. The duration of the blocks ranged from 8.8 to 9.5 s (*M* = 9 s).

#### English word lists

Fifteen English word lists were generated by translating the items in the ASL word lists and additional ASL nouns from the vocabulary corpus. Each word list contained 14 nouns. Each word was presented for 350 ms resulting in a total duration of 4.9 s for each word list.

### ASL sentence comprehension proficiency task

Prior to scanning, each participants' proficiency on ASL sentences (created in the same fashion as the ones presented during fMRI acquisition) was assessed outside the scanner. The proficiency test was comprised of 10 novel ASL sentences and each sentence contained an average of 7 (range = 5–11) ASL words from the vocabulary corpus. Participants were instructed to view each video once and translate the ASL sentence into English on paper provided (see Supplementary Video 1 for an example of an ASL sentence video). Once they completed the test, the subject's translations were given two scores: a semantic and a syntactic score. In the semantic scoring, the vocabulary words from each sentence were scored on whether each word was correctly translated or not. If a word was translated incorrectly, the response was categorized as a semantic error, a phonological error (based on the four parameters of sign language, i.e., hand movement, hand shape, position of hand, and orientation of the sign to the body), or omission. In the syntactic scoring, the translations were scored based on the correct relation of each word to the other word in the sentence (e.g., who was the agent, what is modifying what, etc.). The average number of relations in the sentences was 5 (range = 3–6). Correct scoring of the relations was not dependent on comprehension of lexical items. For example, the ASL sentence in Video 1, translated into English as “My uncle was driving fast and he sped past the woman in the car” has four relations to comprehend: (1) who is driving, (2) what is fast, (3) who sped, and (4) who was sped past. Semantic and syntactic scores were calculated as proportion correct of the number of words and number of relations, respectively, averaged across all sentences.

### fMRI experimental design

The scanning session contained four functional runs with the order counter-balanced across participants. Within each run pseudo-randomized blocks of the four conditions (i.e., ASL sentences, ASL word lists, English sentences, and English word lists) were presented, with rest periods of 12 s interleaved between blocks. In each run, there were equal numbers of blocks from each condition. There were a total of 16 blocks in three of the runs and 12 blocks in one run. After functional scanning, a T1 structural MRI was collected. The whole scanning session lasted approximately 50 min.

### fMRI data acquisition

MRI scanning was performed on a 3T Philips Achieva scanner at the Keller Center for Imaging Innovation at the Barrow Neurological Institute in Phoenix, Arizona. During scanning, participants laid supine and viewed the display through Nordic Neurolab's MR-compatible high-resolution LED goggles. The visual display was synchronized to image acquisition via Neurolab's sync system with E-prime Software Version 2 (Psychology Software Tools, http://www.pstnet.com/). The parameters for the functional runs were as follows: 35 axial-oblique slices (3 mm thickness), in-plane resolution = 3 × 3 mm, *TR* = 2 s, *TE* = 25 ms, flip angle = 80°, FOV = 240 × 240 mm, matrix = 80 × 80, and ascending acquisition. The number of volumes acquired for the four runs were 172, 174, 174, and 132 respectively. The parameters for the high-resolution T1 anatomical scan were as follows: MPRAGE sequence, 170 sagittal slices, TR = 6.742 ms, *TE* = 3.104 ms, flip angle = 9°, matrix = 256 × 256, voxel size = 1.1 × 1.1 × 1.2 mm.

### fMRI data preprocessing and analysis

Image preprocessing and analyses were completed using SPM8 (SPM8, Wellcome Institute of Cognitive Neurology, London, UK). Standard preprocessing steps were implemented, including slice time correction, rigid body motion correction, a high-pass filter at 1/128 Hz to filter low-frequency nonlinear drifts, coregistration of the functional images to each subject's T1 anatomical images, and normalization to the Montreal Neurological Institute (MNI) template. All normalized functional images were smoothed using a Gaussian filter with a full width at half maximum of 8 mm.

Individual subject analyses were conducted by constructing a general linear model for each condition. Four regressors were defined: ASL sentences, ASL word lists, English sentences, and English word lists. For all conditions, the regressors were convolved with a canonical hemodynamic response function (Friston et al., 1994). Voxel-wise repeated measure *t*-tests were performed using the estimated parameters of the regressors (beta weights) to compare across conditions. For group analysis, a random effects analysis was conducted by incorporating the individual data from the first-level analysis of each task. The group results were overlaid onto the averaged normalized anatomical image of the group by using an SPM extension tool, bspmVIEW (http://www.bobspunt.com/bspmview/). Significant clusters were identified using non-parametric permutation and randomization techniques via the SnPM13 toolbox of SPM12 (Nichols and Holmes, 2002; http://warwick.ac.uk/snpm) and a voxel-wise FWE-threshold of *p* < 0.05.

To further investigate how L1 English sentence regions respond to L2 ASL sentences, regions of interest (ROIs) were functionally defined based on the contrast of English sentences–English word lists. The amplitude of the response within each ROI for each condition were then plotted and compared with two-way ANOVAs (language x stimulus type).

Lastly, we conducted correlation analyses to examine the relationship between activation to ASL sentences and an individual's ASL proficiency score. To do so we calculated a Pearson *r*-value in each voxel between the beta values and proficiency scores from each subject. Note that this correlational approach is most likely underpowered in our sample, as power curve estimates computed by Yarkoni and Braver (2010) indicate that a sample size of approximately 50 is needed for sufficient power (0.8) while maintaining a conservative probability of false positives in typical fMRI experiments. Nonetheless we conducted the correlational analyses as this is a difficult population from which to achieve an adequate sample size. Also note that we have used a more relaxed statistical threshold for these results (voxel-wise uncorrected of *p* < 0.005, with a cluster size threshold = 10 voxels).

## Results

### ASL sentence comprehension behavioral measure

**Figure 1:**
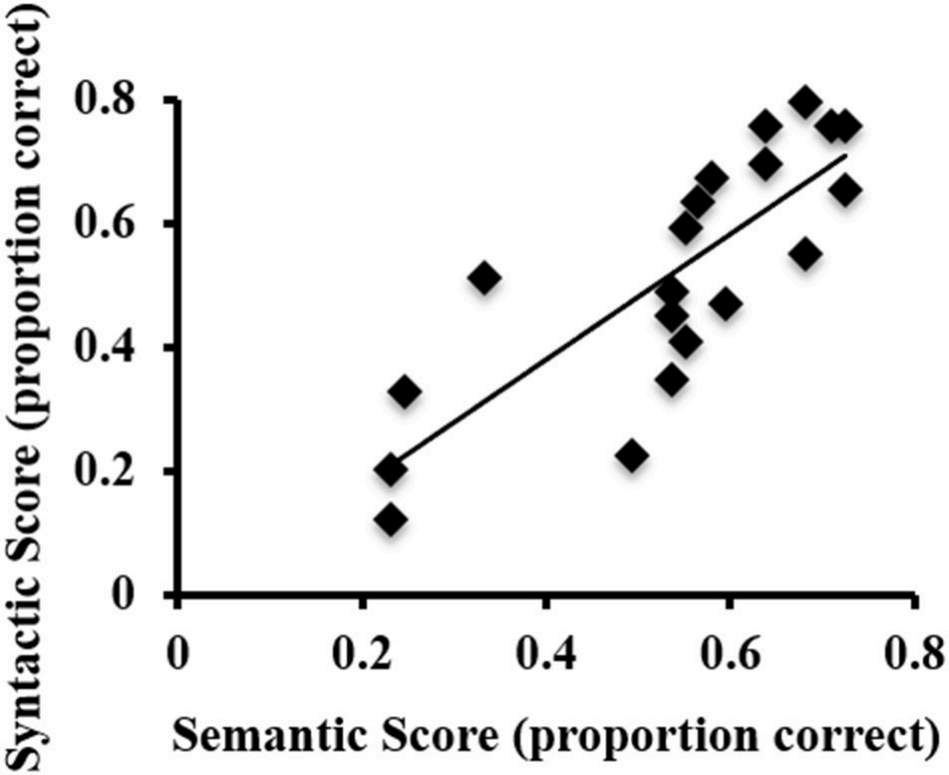
Scatterplot of the syntactic and semantic scores on the behavioral ASL sentence comprehension task for each participant.

ASL sentence comprehension behavioral task performance (proportion correct) was as follows: semantic scores ranged from 0.23 to 0.72 (*M* = 0.54). Syntactic scores ranged from 0.12 to 0.8 (*M* = 0.52). There was a strong positive correlation between the semantic and syntactic proficiency scores, *r* = 0.807, *p* < 0.001 (Figure 1). Thus, in subsequent fMRI analyses that correlate activations with ASL sentence comprehension abilities, an overall proficiency score (i.e., an average of semantic and syntactic scores for each participant) was used.

### fMRI results: L1 English

**Figure 2:**
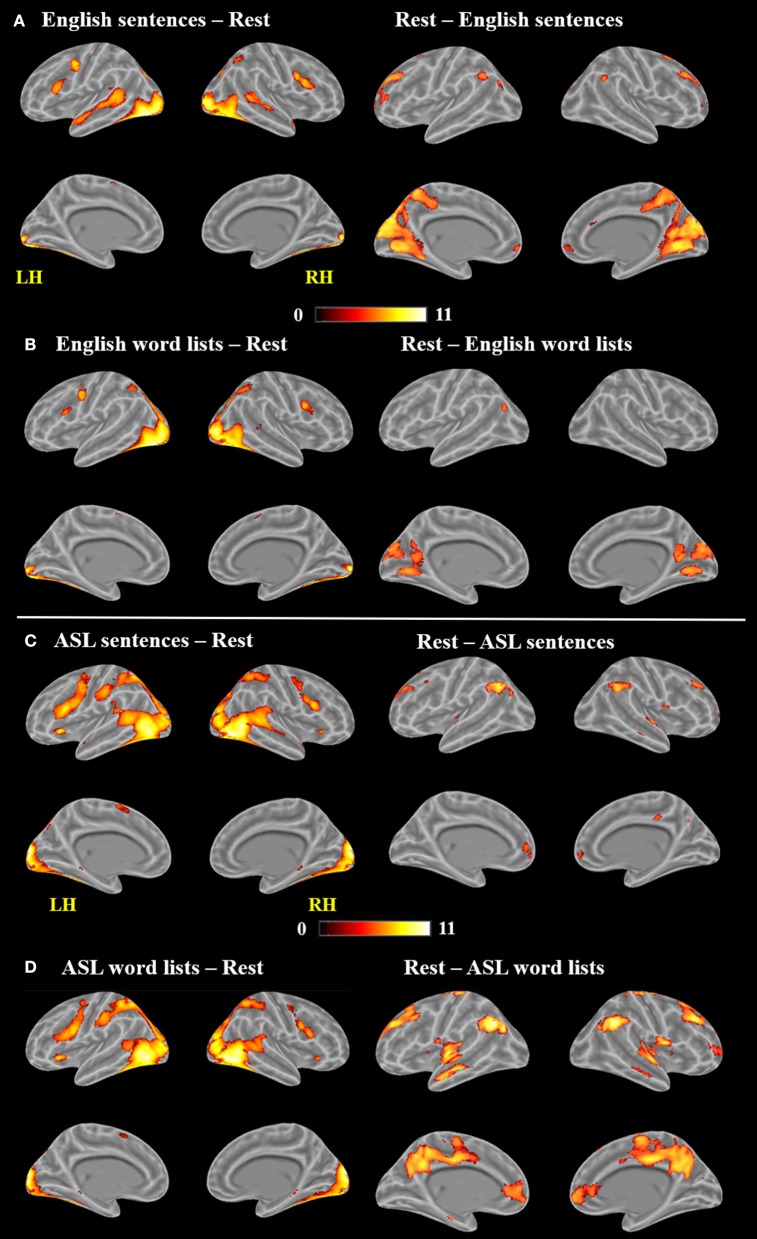
Maps of significant activations for **(A)** English sentences, **(B)** English word lists, **(C)** ASL sentences, and **(D)** ASL word lists compared to rest, FWE corrected, *p* < 0.05.

As observed in previous studies of sentence comprehension (Humphries et al., 2001; Vandenberghe et al., 2002; Rogalsky and Hickok, 2009; Rogalsky et al., 2011), English sentences and word lists each activated large bilateral swaths of cortex in the frontal, temporal and inferior parietal lobes, as well as visual cortex (*p* < 0.05, FWE-corrected Figure 2A, Table 1). For English sentences, widespread significant activations were identified in the right middle frontal gyrus (MFG), right angular gyrus (AG), bilateral precentral gyrus (PrCG), inferior frontal gyri (IFG, predominately in the pars opercularis), superior temporal gyrus (STG), middle temporal gyrus (MTG), intraparietal sulcus (IPS), lingual gyrus, inferior occipital gyrus (IOG), middle occipital gyrus (MOG), fusiform gyrus, calcarine sulcus, and supplementary motor area (SMA). In the English word list condition, significant activations were found in the bilateral IFG (pars opercularis), precentral gyrus, MFG, IOG, MOG, lingual gyrus, fusiform gyrus, and SMA superior parietal lobule (SPL), right MTG, AG, and insula (Figure 2B, Table 1).

**Table 1:** Activation clusters for reading English stimuli.

**Contrast**	**Region**	**Peak coordinates (mm)**			**Cluster size**	**Peak *t*-value**
		**x**	**y**	**z**		
English sentences > Rest	**L IOG**	−**18**	−**92**	−**10**	**3,401**	**11.02**
	L FG	−38	−82	−14		10.90
	L FG	−42	−74	−16		10.79
	**R Calcarine**	**18**	−**94**	−**4**	**3,576**	**9.94**
	R IOG	32	−88	−2		9.63
	R IOG	38	−76	−16		9.52
	L PrCG	−**52**	**0**	**46**	**354**	**8.57**
	R IFG	**42**	**10**	**26**	**642**	**8.55**
	R MFG	44	−2	56		6.28
	R PrCG	54	4	44		5.84
	**L MOG**	−**28**	−**68**	**24**	**164**	**8.23**
	**L MTG**	−**58**	−**46**	**6**	**1,338**	**7.94**
	L MTG	−52	−6	−18		6.91
	L MTG	−56	−32	−2		6.88
	**L SMA**	**0**	**6**	**64**	**220**	**7.48**
	**L IFG**	−**52**	**18**	**22**	**423**	**7.13**
	**R MOG**	**32**	−**66**	**26**	**103**	**6.50**
	**R AG**	**34**	−**56**	**50**	**192**	**6.38**
Rest > English sentences	**L Cuneus**	−**14**	−**80**	**40**	**11,432**	**9.15**
	R Cuneus	8	−82	38		8.83
	L Cuneus	−12	−74	26		8.82
	**L SFG**	−**28**	**38**	**36**	**963**	**7.22**
	L SFG	−26	56	22		5.54
	L SFG	−24	56	4		5.39
	**R MFG**	**30**	**32**	**36**	**353**	**6.41**
	R MFG	28	46	32		5.52
	**L AG**	−**52**	−**56**	**38**	**189**	**6.31**
	**R SFG**	**20**	**16**	**56**	**128**	**5.98**
	**L MOG**	−**38**	−**80**	**32**	**66**	**5.94**
	**L Cingulate**	**0**	**28**	**28**	**843**	**5.90**
	L MeFG	0	56	−2		5.90
	R Cingulate	2	44	6		5.06
	**R AG**	**54**	−**54**	**36**	**98**	**5.71**
	**R MFG**	**30**	**58**	**4**	**106**	**5.70**
	**L STG**	−**40**	−**18**	−**4**	**33**	**5.65**
	**Pons**	−**2**	−**10**	−**24**	**12**	**5.47**
English word lists > Rest	**L FG**	−**36**	−**82**	−**16**	**4,394**	**11.47**
	L LG	−16	−94	−10		10.05
	L MOG	−34	−88	−2		10.00
	**R Calcarine**	**16**	−**92**	−**4**	**3,556**	**10.51**
	R IOG	34	−84	0		10.44
	R FG	40	−74	−16		10.18
	**L PrCG**	−**52**	−**2**	**46**	**255**	**8.64**
	**R IFG**	**44**	**8**	**28**	**411**	**8.19**
	R PrCG	56	6	40		5.45
	L SMA	**0**	**2**	**62**	**258**	**6.59**
	**R MOG**	**32**	−**66**	**26**	**516**	**6.56**
	R AG	34	−54	48		6.43
	**L IFG**	−**42**	**8**	**22**	**109**	**5.93**
	**R MFG**	**48**	−**4**	**54**	**28**	**5.66**
	**R MTG**	**48**	−**42**	**8**	**27**	**5.18**
	**L PrCG**	−**34**	−**6**	**46**	**17**	**5.07**
	**R Insula**	**34**	**26**	**4**	**9**	**4.83**
	**R IFG**	**52**	**34**	**18**	**5**	**4.78**
Rest > English word lists	**R LG**	**0**	−**72**	−**4**	**4,793**	**7.73**
	L Cuneus	2	−78	26		6.95
	R Cuneus	8	−82	38		6.94
	**L MOG**	−**44**	−**76**	**28**	**149**	**5.76**
	**L Cingulate**	−**2**	**42**	−**6**	**39**	**5.11**
	**R STG**	**42**	−**16**	−**2**	**4**	**4.77**
English sentences > Word lists	**L MTG**	−**54**	−**6**	−**18**	**1,248**	**7.65**
	L MTG	−58	−38	2		7.53
	L MTG	−56	−16	−10		7.01
	**R MTG**	**60**	**0**	−**16**	**174**	**6.86**
	**L MeFG**	−**8**	**58**	**34**	**16**	**5.77**
	**L IFG**	−**54**	**20**	**16**	**41**	**5.59**
	**L STG**	−**42**	**24**	−**20**	**24**	**5.05**
	**R MTG**	**50**	−**36**	**0**	**6**	**4.88**
English word lists > Sentences	**L MOG**	−**24**	−**90**	**16**	**898**	**7.60**
	L Calcarine	−10	−88	0		5.87
	L SOG	−18	−86	32		5.81
	**R MOG**	**26**	−**80**	**16**	**209**	**6.44**
	**R IPL**	**58**	−**40**	**48**	**184**	**6.41**
	**R Cingulate**	**10**	**22**	**28**	**137**	**5.50**
	R Cingulate	4	32	26		5.10
	**L FG**	−**26**	−**70**	−**14**	**16**	**4.88**
	**L Cingulate**	−**8**	**24**	**26**	**3**	**4.84**
	**R Calcarine**	**12**	−**86**	**0**	**2**	**4.77**

*Coordinates are reported in MNI space. The bold text indicates the peak voxel for that cluster. All clusters listed are significant at FWE-corrected p < 0.05. Regions: angular gyrus (AG); fusiform gyrus (FG); inferior frontal gyrus (IFG); inferior occipital gyrus (IOG); inferior parietal lobule (IPL); lingual gyrus (LG); middle frontal gyrus (MFG); medial frontal gyrus (MeFG); middle occipital gyrus (MOG); middle temporal gyrus (MTG); precentral gyrus (PrCG); superior frontal gyurs (SFG); superior temporal gyrus (STG); supplementary motor area (SMA); superior occipital gyrus (SOG)*.

Clusters more active for rest than the English sentences were identified bilaterally in the superior frontal gyrus (SFG), AG, cuneus, cingulate gyrus, medial frontal gyrus (MeG) and pons, in the left MOG and STG, and in the R MFG (Figure 2A; Table 1). Clusters more active for rest than the English word lists were found in the bilateral CG, cuneus and medial occipital lobe, including LG and MOG, as well as a small cluster (4 voxels) in the right STG (Figure 2B; Table 1).

**Figure 3:**
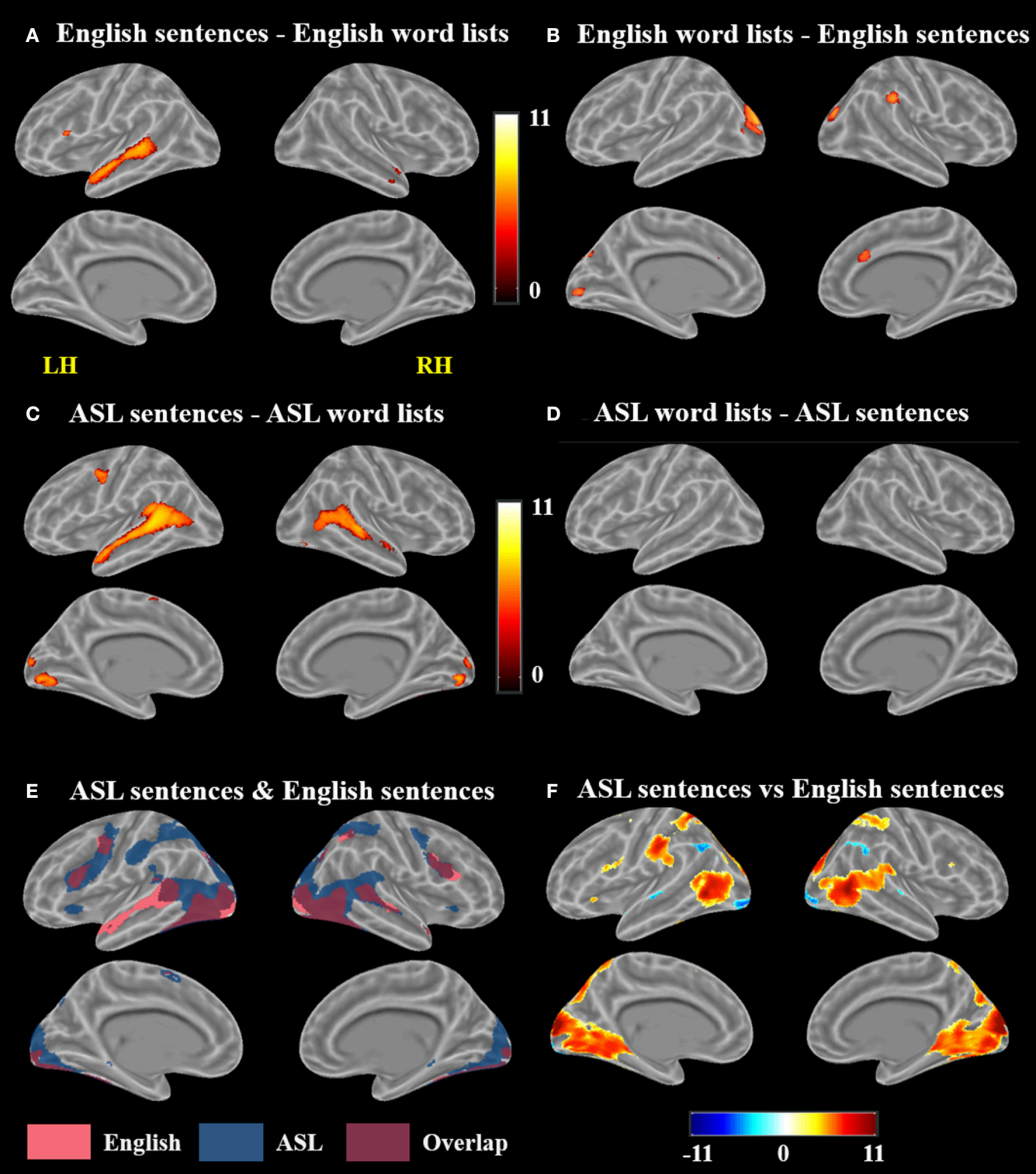
Contrast results for **(A)** English sentences > English word lists, **(B)** English word lists > English sentences, **(C)** ASL sentences > ASL word lists, **(D)** ASL word lists > ASL sentences but no voxels survived correction, **(E)** conjunction of ASL sentences > rest and English sentences > rest, and **(F)** ASL sentences vs. English sentences; in **(F)**, warmer colors indicate greater activation for ASL sentences and cooler colors indicate greater activation for English sentences (FWE corrected, *p* < 0.05).

A voxel-wise *t*-test of English sentences vs. English word lists revealed increased activation for English sentences compared to word lists in the bilateral temporal lobes in the STG and MTG (right anterior temporal lobe, left activation spanned the length of the temporal lobe), as well as the left IFG (pars opercularis and pars triangularis) and MeG (Figure 3A; Table 1). Clusters more active for the English word lists than the English sentences were identified in bilateral CG and occipital regions including MG, SOG, and FG, as well as in the right inferior parietal lobule (Figure 3B).

### fMRI results: L2 ASL

Large swaths of activations were found in response to ASL sentences compared to rest in the bilateral visual cortex, extending both into posterior superior temporal and inferior parietal cortex, as well as the SPL and postcentral gyrus. Bilateral frontal regions including IFG (pars orbitalis, opercularis and triangularis), MFG, precentral gyrus and SMA also contained significant activations, in addition to bilateral hippocampus and left pallidum (*p* < 0.05, FWE-corrected, Figure 2C, Table 2). ASL word lists compared to rest activated a similar set of regions as the ASL sentences, including bilateral SPL, IPL, cuneus,PrCG, IFG, MFG, insula, SOG, inferior temporal gyrus (ITG), FG, hippocampus, and SMA (Figure 2D, Table 2).

**Table 2:** Activation clusters for viewing ASL stimuli.

**Contrast**	**Region**	**Center coordinates (mm)**			**Cluster size**	**Peak *t*-value**
		**x**	**y**	**z**		
ASL sentences > Rest	**R ITG**	**48**	−**70**	−**8**	**19,252**	**11.60**
	R Calcarine	12	−94	12		11.60
	L SOG	−8	−98	8		11.37
	R Hippocampus	**18**	−**30**	−**4**	**898**	**10.02**
	L Hippocampus	−20	−30	−6		8.47
	**R IFG**	**42**	**12**	**24**	**803**	**9.39**
	R MFG	44	−2	56		7.17
	**L Insula**	−**28**	**28**	−**2**	**279**	**9.21**
	**L SMA**	−**2**	**10**	**56**	**498**	**7.42**
	R IPL	**32**	−**52**	**56**	**762**	**7.26**
	R SMaG	32	−38	46		6.10
	**R Insula**	**32**	**28**	−**2**	**106**	**6.95**
	**R MTG**	**54**	**10**	−**20**	**45**	**5.89**
	**L MTG**	−**56**	**0**	−**18**	**61**	**5.71**
	**L STG**	−**50**	**16**	−**18**	**7**	**4.94**
	**L Pallidum**	−**18**	**8**	**2**	**1**	**4.62**
Rest > ASL sentences	**L AG**	−**50**	−**62**	**40**	**700**	**8.90**
	**R AG**	**58**	−**54**	**36**	**607**	**8.54**
	R AG	52	−66	30		6.25
	R AG	46	−66	42		5.51
	**R STG**	**52**	−**6**	**2**	**529**	**7.51**
	R STG	42	−16	−2		5.18
	**L Hippocamus**	−**18**	−**42**	**12**	**236**	**7.04**
	L Hippocampus	−32	−42	−2		5.15
	**R Hippocampus**	**22**	−**40**	**12**	**209**	**7.00**
	**R MTG**	**66**	−**20**	−**12**	**36**	**6.26**
	**L MFG**	−**26**	**40**	**32**	**293**	**6.00**
	L MFG	−22	52	28		5.67
	**R Putamen**	**30**	−**12**	**0**	**83**	**5.99**
	**R MFG**	**30**	**32**	**36**	**133**	**5.97**
	**L STG**	−**40**	−**20**	−**2**	**67**	**5.90**
	**L MeFG**	**0**	**56**	**0**	**484**	**5.88**
	**R MFG**	**28**	**58**	**4**	**76**	**5.67**
	**R Cingulate**	**2**	−**28**	**40**	**74**	**5.18**
	L Cingulate	0	−42	44		4.67
ASL word lists > rest	**L SOG**	−**10**	−**98**	**10**	**17,795**	**11.91**
	R ITG	48	−68	−6		11.86
	R Cuneus	10	−94	14		11.74
	**L Hippocampus**	−**20**	−**30**	−**4**	**858**	**10.06**
	R Hippocampus	20	−30	−4		9.91
	R LG	6	−30	−8		6.23
	**L PrCG**	−**48**	**6**	**20**	**1,827**	**9.34**
	L PrCG	−30	−6	50		6.54
	L PrCG	−44	−2	54		6.32
	**L Insula**	−**30**	**28**	−**2**	**331**	**8.70**
	**R IFG**	**46**	**10**	**26**	**724**	**7.90**
	R MFG	44	−2	56		6.85
	R PrCG	56	6	40		6.20
	**L SMA**	−**2**	**10**	**54**	**322**	**7.23**
	**R Insula**	**34**	**28**	−**4**	**118**	**6.58**
	**R IFG**	**32**	**38**	−**18**	**4**	**4.98**
	**R SMaG**	**64**	−**20**	**38**	**2**	**4.83**
Rest > ASL word lists	**R AG**	**56**	−**54**	**38**	**980**	**10.91**
	R AG	50	−66	34		9.36
	**R Hippocampus**	**20**	−**40**	**12**	**14,168**	**10.70**
	L STG	−38	−22	−2		10.48
	L Precuneus	0	−54	40		10.34
	**L MOG**	−**44**	−**72**	**36**	**1,064**	**10.43**
	**R MFG**	**32**	**32**	**40**	**1,358**	**9.19**
	R MFG	24	20	54		7.44
	R SFG	16	32	54		6.54
	**L MFG**	−**28**	**38**	**34**	**1,768**	**9.13**
	L MFG	−38	20	42		7.16
	L MFG	−30	22	40		6.97
	**R MeFG**	**2**	**54**	**0**	**2,118**	**8.34**
	L Cingulate	0	38	4		7.00
	R MFG	28	58	4		6.81
	**L Hippocampus**	−**16**	−**18**	−**22**	**130**	**7.48**
	**L Caudate**	−**18**	**4**	**24**	**227**	**6.06**
	L Caudate	−16	−10	24		5.77
ASL sentences > Word lists	**L MTG**	−**58**	−**46**	**8**	**2,206**	**8.00**
	LMTG	−54	−40	4		7.94
	L MTG	−54	−4	−18		7.74
	**R MTG**	**48**	−**34**	**0**	**1,436**	**7.52**
	R MTG	48	−42	8		6.59
	R MTG	56	−8	−14		6.15
	**L Calcarine**	**2**	−**86**	−**8**	**1,032**	**7.29**
	L Calcarine	−4	−96	6		5.95
	R Calcarine	10	−94	6		5.24
	**L PrCG**	−**46**	**0**	**50**	**206**	**6.26**
	L PoCG	−54	−6	44		6.07
	**R FG**	**42**	−**56**	−**22**	**54**	**6.04**
	**L SMA**	−**2**	**4**	**62**	**140**	**5.84**
	**R FG**	**28**	−**78**	−**16**	**74**	**5.58**
	**R IOG**	**46**	−**76**	−**14**	**11**	**5.15**
	**L Precuneus**	**0**	−**58**	**46**	**26**	**4.96**

*Coordinates are reported in MNI space. The bold text indicates the peak voxel for that cluster. All clusters listed are significant at voxel-wise FWE p < 0.05. Regions: fusiform gyrus (FG); inferior frontal gyrus (IFG); inferior occipital gyrus (IOG); inferior pariatel lobule (IPL); inferior temporal gyrus (ITG); lingual gyrus (LG); middle frontal gyrus (MFG); medial frontal gyrus (MeFG); middle temporal gyrus (MTG); postcentral gyrus (PoCG); precentral gyrus (PrCG); superior occipital gyrus (SOG); superior temporal gyrus (STG); supplementary motor area (SMA); supramarginal gyrus (SMaG)*.

Clusters more active during rest than ASL sentences were identified in the bilateral AG, STG, MTG, CG, MFG, MeFG, hippocampus, and insula, as well as the right putamen (Figure 2C, Table 2). Clusters more active during rest than ASL word lists include portions of bilateral STG, Heschl's gyrus, AG, MFG, SFG, MeG, and CG, precuneus, as well as left caudate (Figure 2D, Table 2).

A voxel-wise *t*-test of ASL sentences vs. ASL word lists identified clusters in bilateral STG, MTG, SMA, in occipital cortex with peaks in FG, IOG, precuneus and in the calcarine sulcus, as well as in the left pre and postcentral gyri (Figure 3C, Table 2). No regions were more active in response to ASL word lists than ASL sentences (Figure 3D).

### fMRI results: L2 ASL vs. L1 English

A conjunction map of the brain regions significantly activated by ASL sentences and by English sentences was generated to describe shared vs. distinct sentence processing regions for the two types of sentences (Figure 3E). Areas of overlap were found bilaterally in the frontal lobe (bilateral IFG–predominately pars opercularis, MFG and precentral gyrus), posterior superior and middle temporal gyri, and bilateral occipital visual cortex. Notably the spatial extent of activation was greater for ASL sentences than English sentences in the inferior parietal lobe, superior parietal lobule, and inferior frontal gyrus. The response to English sentences compared to ASL sentences extended more anteriorly along the left superior temporal sulcus, although ASL sentences did elicit significant activation in a small cluster in the left anterior temporal lobe (45 voxels) (Figure 3E).

To further examine the differences between the neuroanatomy supporting the comprehension of ASL sentences and English sentences, we also directly compared the activation between these two conditions in a voxel-wise *t*-test (Figure 3F; Table 3). ASL sentences yielded significantly greater activation than English sentences in bilateral SPL, occipital-inferior temporal cortex including SOG, ITG and the calcarine sulcus, IFG (both pars opercularis and pars triangularis in the left hemisphere), insula, and SMA, as well as in the left PrCG, and right hippocampus. English sentences yielded significantly greater activation than ASL sentences in bilateral MTG, AG, IPL, and occipital regions including peaks in the LG and IOG (Figure 3F; Table 3).

**Table 3:** Activation clusters for the contrast between ASL sentences and English sentences.

**Contrast**	**Region**	**Center coordinates (mm)**			**Cluster size**	**Peak *t*-value**
		**x**	**y**	**z**		
ASL sentences > English sentences	**R Calcarine**	**10**	−**94**	**12**	**15,592**	**13.81**
	L SOG	−8	−98	10		13.59
	R Hippocampus	18	−30	−6		11.20
	**L MOG**	−**50**	−**70**	**2**	**1,538**	**10.01**
	**L IFG**	−**50**	**8**	**18**	**231**	**6.55**
	**L ITG**	−**44**	−**38**	−**22**	**63**	**5.86**
	**L Insula**	−**28**	**30**	−**2**	**64**	**5.76**
	**L PrCG**	−**28**	−**6**	**56**	**145**	**5.67**
	**L SMA**	−**2**	**14**	**52**	**53**	**5.49**
	**R IFG**	**40**	**12**	**22**	**15**	**5.08**
	**L SMA**	−**14**	**4**	**66**	**1**	**4.76**
	**R Insula**	**32**	**28**	−**2**	**2**	**4.70**
English sentences > ASL sentences	**R IOG**	**24**	−**98**	−**6**	**146**	**7.55**
	R IOG	28	−92	−10		7.27
	**L LG**	−**20**	−**94**	−**12**	**259**	**6.71**
	L IOG	−32	−92	−12		6.55
	**R AG**	**58**	−**56**	**34**	**260**	**6.52**
	R AG	46	−66	42		6.00
	R IPL	54	−56	42		5.69
	**L AG**	−**48**	−**70**	**36**	**315**	**6.48**
	**R MTG**	**66**	−**22**	−**6**	**72**	**6.26**
	**L MTG**	−**64**	−**22**	−**8**	**191**	**6.20**

*Coordinates are reported in MNI space. The bold text indicates the peak voxel for that cluster. All clusters listed are significant at voxel-wise FWE p < 0.05. Regions: angular gyrus (AG); inferior frontal gyrus (IFG); inferior occipital gyrus (IOG); inferior parietal lobule (IPL); inferior temporal gyrus (ITG); lingual gyrus (LG); middle occipital gyrus (MOG); middle temporal gyrus (MTG); precentral gyrus (PrCG); superior occipital gyrus (SOG); supplementary motor area (SMA)*.

### Response of L1 English regions to L2 ASL sentences

**Figure 4:**
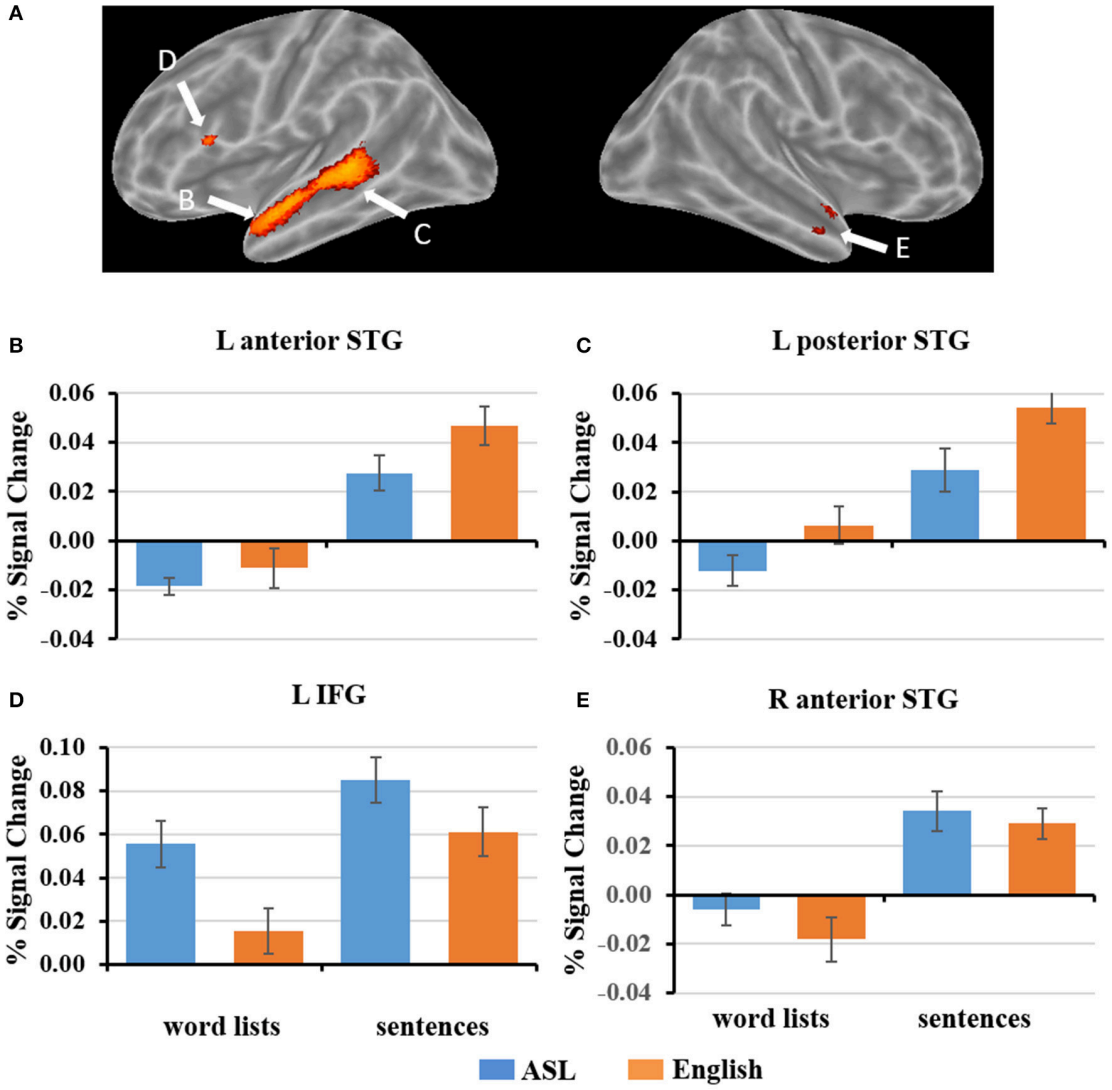
**(A)** ROIs defined by English sentences > English word lists (FWE corrected, *p* < 0.05). **(B–E)** Graphs of the response to each condition of select ROIs depicted in **(A)**. Error bars represent standard error of the mean.

To explore how ASL sentences engage English sentence processing regions, we plotted the mean percent signal change during each condition for the regions identified to be significantly more activated for English sentences than word lists, (*p* < 0. 05, FWE-corrected). As described in section fMRI Results: L2 ASL vs. L1 English, the regions identified were in the left inferior frontal gyrus, left anterior and posterior superior temporal gyrus, and right anterior temporal gyrus (Figure 4). 2 × 2 ANOVAs were computed for each ROI. The results are as follows (α = .05): The left anterior temporal ROI (Figure 4B) exhibited a significant main effect of sentence structure, *F*_(1, 19)_ = 67.4, *p* < 0.001; English and ASL sentences exhibit greater activation than word lists in their respective modality. The main effect of language was not significant, *F*_(1, 19)_ = 3.5, *p* = 0.07. There was no significant interaction in the left anterior temporal ROI [*F*_(1, 19)_ = 1.75, *p* = 0.20]. The left posterior STG ROI (Figure 4C) exhibited a main effect for stimulus type [sentences >word lists; *F*_(1, 19)_ = 98.7, *p* < 0.001], and main effect for modality [English >ASL, *F*_(1, 19)_ = 7.5, *p* = 0.01]; the interaction was not significant (*p* = 0.24). These findings suggest that this left posterior temporal region is sensitive to sentence structure in both L1 and L2, but with a preference for L1. The left IFG ROI (Figure 4D) exhibited a significant main effect for sentence structure [*F*_(1, 19)_ = 28.2, *p* < 0.001] with sentences activating the ROI more than word lists, as well as a significant main effect of language [*F*_(1, 19)_ = 18.8, *p* < 0.001], with ASL significantly activating more than English; the interaction was not significant (*p* = 0.23). The right anterior temporal ROI (Figure 4E) exhibited a significant main effect for sentences than word lists, [*F*_(1, 19)_ = 54.3, *p* < 0.001], but no significant main effect of language (*p* = 0.21) or interaction (*p* = 0.33). To summarize these ROI results, no ROIs showed an interaction, only the L pSTG ROI was significantly activated more for English than ASL, and the reverse was found in the L IFG, in which ASL led to greater activation than English. All of the ROIs were significantly more activated for sentences than word lists. These findings suggest that L1 English sentence comprehension networks are also engaged during L2 ASL sentence comprehension, albeit to varying degrees.

### Exploratory fMRI results: ASL proficiency

To determine whether the engagement of English sentence processing regions during ASL sentence comprehension is dependent on level of ASL proficiency, we conducted correlation analyses to examine the relationship between activation to ASL sentences and an individual's ASL proficiency score. Although this approach is likely underpowered in our sample (Yarkoni and Braver, 2010), we nonetheless present results as this is a difficult population from which to achieve an adequate sample size. The following results are therefore exploratory in nature and null results should be interpreted with great caution.

**Figure 5:**
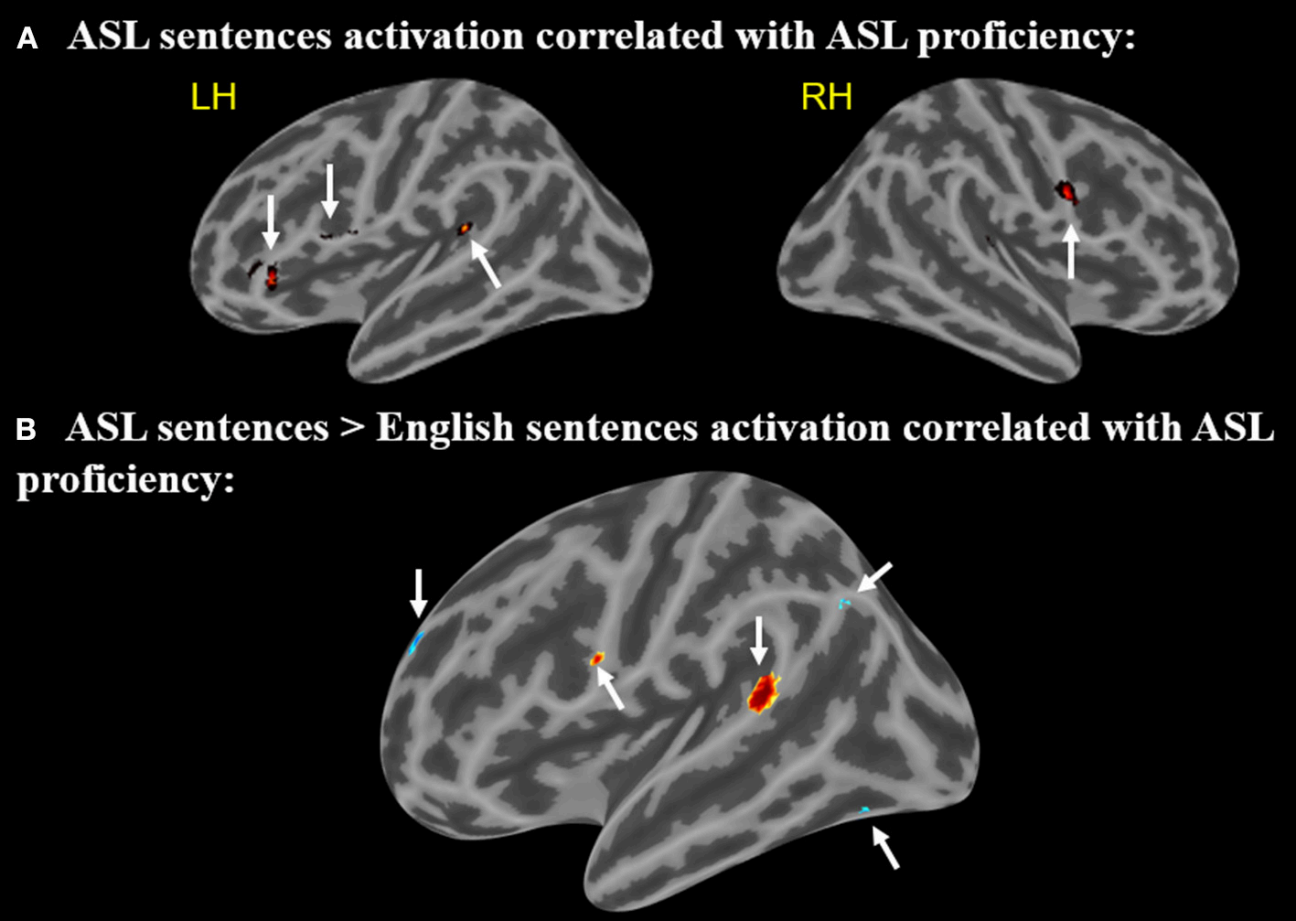
**(A)** Regions in which activation to ASL sentences > rest is correlated with ASL proficiency. Arrows point to regions with activations that positively correlate with ASL proficiency **(B)** Regions in which activation to ASL sentences > English sentences is correlated with ASL proficiency. Warmer colors indicate a positive correlation, cooler colors indicate a negative correlation (voxel-wise uncorrected *p* <.005, cluster size threshold = 10 voxels). Arrows **(A,B)** point to regions with activations that positively correlate with ASL, arrows **(C–E)** point to regions that negatively correlate.

A positive correlation between ASL proficiency score and activation in response to ASL sentences compared to rest was found in the left IFG (pars opercularis) (peak *r* = −58 14 26), left IFG (pars orbitalis) (−44 34 −2), left posterior STG (−46 −40 16), and the right MFG (58 4 28) (*p* < 0.005, minimum cluster size = 10 voxels Figure 5A). These positive correlations suggest that the activation of these regions while viewing ASL sentences is associated with increased levels of ASL proficiency. Note that all of these regions identified as related to ASL proficiency are a subset of those found to be activated by English sentences compared to rest.

A second correlational analysis was conducted to focus more specifically on ASL sentence comprehension; a correlation was computed for the relationship between greater activation to ASL sentences compared to English sentences and an individual's ASL proficiency score. Positive correlations between ASL proficiency and activation in response to ASL sentences (minus English sentences) were observed in the left IFG (pars opercularis) (−58 6 22) and left posterior STG (−64 −42 14) (Figure 5B). A negative correlation in the left temporal-occipital gyrus (extending into the fusiform gyrus) (−38 −68 −14), left superior frontal gyrus (−14 60 28), and left supramarginal/angular gyrus (−46 −68 38) (Figure 5B) was also observed. These negative correlations indicate that greater activation for ASL vs. English in these regions is associated with decreased levels of ASL proficiency. The regions with positive correlations to ASL proficiency also are activated by English sentences, but the regions with negative correlations do not overlap with the activations to English sentences.

## Discussion

The present study investigated the brain regions involved in sentence processing of ASL in novice L2 adult hearing signers. Our aim was to determine how L1 spoken language sentence processing networks respond to L2 ASL sentences. Overall our results combined with previous work indicate that ASL syntactic processing engages brain regions that are highly overlapping with typical spoken language L1 language networks, independent of proficiency or age of acquisition. In other words, modality alone does not substantially modulate the location of the neural substrates of sentence processing resources, which leads us to conclude that spoken L1 language processing networks can quickly adapt to L2 syntactic structure that is highly unrelated to the syntactic structure of L1.

These findings coincide with separate previous functional neuroimaging studies of spoken language bilingualism and early bimodal bilingualism, suggesting that age of acquisition and modality may not necessarily impact macro neural organization of L2 (Abutalebi et al., 2001; Emmorey et al., 2016). However, each of the L1 sentence comprehension regions were modulated differently by L2 ASL vs. L1 English sentences, providing valuable insights into the nature of each region's contributions to L1 sentence processing (discussed below). Our exploratory analyses of ASL L2 proficiency indicate that greater ASL proficiency is positively correlated with greater activation of both left frontal and posterior temporal sentence processing regions, and negatively correlated with activation in visual and visual-spatial processing regions. The implications of our findings regarding the response properties and flexibility of sentence-processing networks are discussed below.

### Contributions of age of acquisition and proficiency vs. modality to ASL sentence processing

In the present study alone, it is not possible to differentiate between the contributions of age of acquisition, proficiency, and language modality to our findings. However, the relative wealth of literature in spoken language bilingualism investigating effects of age of acquisition and proficiency, and (to a lesser degree) in early bimodal bilingualism investigating modality effects in the neural correlates of sentence processing, allows us to interpret our results in a meaningful way to better understand the nature and response properties of sentence processing regions.

In studies of early bimodal bilinguals, there is a high degree of overlap between the neural correlates of sign and spoken sentence comprehension (Neville et al., 1997; Bavelier et al., 1998) suggesting that the neural resources supporting sentence comprehension are largely independent of modality. In our late L2 ASL signers, we also find a high degree of overlap, particularly in inferior frontal and posterior temporal cortex, suggesting that the functional plasticity of spoken language sentence processing regions also is not dependent upon an early age of acquisition and/or high degree of proficiency. Based on previous work in spoken language bilinguals, we expect this overlap to further increase as proficiency improves, even in late L2 learners (Rossi et al., 2006; Tanner et al., 2013).

The overlap we found in bilateral superior temporal cortex for sentence-specific processing (vs. word lists) in both L1 English and L2 ASL, particularly in pSTG, also speaks to the adaptability of these well-investigated L1 sentence processing regions. Previous work in spoken languages has demonstrated that they are engaged during sentence processing regardless of age of acquisition or proficiency (Perani et al., 1998; Jeong et al., 2007), and bilateral STG involvement also has been found in ASL acquired from an early age (Neville et al., 1997). Our study suggests that even in late L2 learning, bilateral superior temporal cortex is responsive to syntactic structures in a different modality.

The age of L2 acquisition is quite similar across our participants, as they all were young adult undergraduates. Thus, any differences detected in our exploratory proficiency correlational analyses are likely due to proficiency differences, not age of acquisition. While there is some variability in proficiency in our sample (Figure 1), certainly future longitudinal studies are needed to better understand how L2 proficiency would affect our findings. But, within our sample, an interesting pattern of activations in the left hemisphere was found to correlate with ASL proficiency: greater activations to ASL sentences than English sentences in regions responsive to English sentences in Broca's area and the pSTG (i.e., regions activated by English sentences in the present study and in numerous previous studies) were associated with greater ASL proficiency. Conversely, greater activation to ASL sentences compared to English sentences in visual-spatial occipital and parietal left hemisphere regions were significantly correlated with lower ASL proficiency. This intra-hemispheric difference in contributions of visual-spatial regions and L1 language regions as a function of ASL proficiency in our late L2 signers suggests that their ASL exposure may be sufficient to expand the response properties of L1 spoken language regions to L2 ASL. The alternative explanation would be that our more proficient ASL signers naturally engage L1 language regions in response to ASL even prior to the development of ASL proficiency. However, this explanation is unlikely to be correct: previous work indicates that in response to ASL vs. non-linguistic biological motion, non-signers exhibit significantly more activation in visual processing regions such as the occipito-temporo-parietal junction, whereas signer significantly more activate known peri-sylvian language regions (Malaia et al., 2012).

In the next three sections, we review in more detail the findings in the three regions most frequently implicated in sentence processing in the neurobiology of language literature more generally.

### Broca's area

A portion of Broca's area, the pars opercularis, was found to be more activated by English sentences compared to English word lists. This finding coincides with numerous previous neuroimaging studies of L1 spoken languages that report greater Broca's area activation for reading sentences than word lists (Fedorenko et al., 2010, 2011, 2012a,b,c; Fedorenko and Kanwisher, 2011; Blank et al., 2016), as well as with previous neuroimaging and electrophysiological studies of L1 ASL sentence comprehension (Neville et al., 1997; Newman et al., 2002, 2010a). The exact nature(s) of the role(s) of Broca's area in sentence comprehension remains highly debated, with prominent hypotheses suggesting syntactic-specific processes (Grodzinsky, 2000; Grodzinsky and Santi, 2008), verbal working memory (Just et al., 1996; Kaan and Swaab, 2002; Rogalsky et al., 2008; Pettigrew and Hillis, 2014), hierarchical structure building (Friederici, 2009; Makuuchi et al., 2012), and cognitive control (Novick et al., 2005) as possible candidates. The present dataset may provide valuable insights into this debate: our ROI plots indicate that the portion of Broca's area engaged in English sentence comprehension (i.e., English sentences >English word lists) also shows preference for ASL sentences compared to word lists. In fact, the English sentence ROI in Broca's area is significantly more activated by ASL sentences than English sentences (Figure 4D). This increased activation for ASL sentences compared to English sentences suggests that this region is not contributing via syntactic movement-specific resources (e.g., Grodzinsky and Santi, 2008), coinciding with previous findings within spoken languages (Rogalsky et al., 2015), but rather this portion of Broca's area is more likely contributing to sentence processing as a cognitive resource, which likely would be taxed more due to increased processing demands to comprehend the less familiar L2 ASL sentences. ASL sentences also activated a cluster in the right hemisphere anatomical homolog of Broca's area, the right IFG, significantly more than English sentences. The right IFG is frequently implicated in L2 spoken language processing (Sebastian et al., 2011; Wei et al., 2015), and increased right IFG activation during language tasks more generally is thought to reflect an increase in cognitive demands and effortful processing (Prat and Just, 2010; Prat et al., 2011; Gan et al., 2013; Mack et al., 2013).

In our exploratory analysis of neural correlates of L2 ASL proficiency, we found that greater activation in Broca's area is significantly correlated with greater ASL proficiency. At first glance, this finding may seem counter to previous findings that IFG involvement is associated with lower L2 proficiency (e.g., Rüschemeyer et al., 2005, 2006). We suspect that the previously-found scenario would be more likely in the present study if our sample included a greater range of ASL proficiency. However, our participants were all novice ASL learners who all first began to have substantial exposure to ASL in adulthood; it is possible that in our sample the participants who engaged in more effortful processing of the ASL stimuli also may engage more effortful processing during ASL instruction, thereby leading to relatively higher proficiency. This idea would align with Leonard et al.'s (2013) finding of greater left and right IFG activity for ASL words than written or spoken English words amongst the top achievers in the ASL classes they sampled from, and the finding of Williams et al. (2016) that greater activation during an ASL phoneme categorization task in Broca's area was seen after 45 h of ASL instruction compared to before any ASL instruction. While the present and previous findings of a greater response in Broca's area postively correlating with L2 proficiency could be due to intrinsic, individual differences that support greater L2 learning success, it is more likely that these findings reflect involvement of Broca's area in the learning of syntactic rules. This explanation coincides with findings in novel languages: IFG has been found to be activated during the successful learning of syntactic rules in artificial grammars (Opitz and Friederici, 2004; Friederici et al., 2006). Bilingualism also does require a degree of cognitive control between the two languages (Kroll and Tokowicz, 2005; Abutalebi, 2008). Our relatively novice L2 ASL learners may require even greater control than a fluent bilingual, thus these Broca's area activations may reflect more effortful and less automatic processing of the second language (Friederici, 2006). As a learner becomes more proficient (beyond the 1–2 years of coursework taken by our participants), the movement between ASL and English may become more automatic or fluid, and thus the need for cognitive control may decline (Emmorey et al., 2008). A longitudinal study would be needed to track this possible rise and fall of Broca's area involvement in L2 ASL sentence processing.

### Anterior temporal lobe

Our analyses implicate the ATL more in the perception of both L2 ASL and L1 English sentences then their respective word list comparison conditions (Figures 3A,C). These findings coincide with numerous previous functional neuroimaging studies of spoken languages in which greater ATL activation to reading and hearing sentences was seen in comparison to unstructured lists of words (Humphries et al., 2001; Vandenberghe et al., 2002; Rogalsky and Hickok, 2009; Rogalsky et al., 2011). Lesion studies also implicate the left anterior temporal lobe in sentence comprehension (in addition to left posterior temporal cortex, discussed below) (Thothathiri et al., 2012; Magnusdottir et al., 2013; Pillay et al., 2014)^2^. Previous studies of both deaf and hearing native ASL signers also have found the ATL to be sensitive to ASL sentence structure (Neville et al., 1997; cf. MacSweeney et al., 2006; Newman et al., 2010b).

Established sentence-processing regions of the ATL adapt to the new modality's sentence structures: the bilateral ATL ROIs defined by the contrast of English sentences–English words both were found to have a main effect of structure, with sentences activating these regions more than word lists (not surprisingly, at least for English, given the defining contrast). It is notable though that in the right ATL ROI, there was no main effect for language, and in the left ATL ROI the main effect for language was not significant but trending toward a preference for English (*p* = 0.07; Figures 4B,E). These findings suggest that L1 sentence-processing regions in the bilateral ATL do adapt to L2 ASL sentence structure, even in late L2 learners.

It is worth mentioning that the right ATL sentence ROI is more anterior than the left ATL sentence ROI, thus it is possible that the right ROI's response reflects language-related semantic processes in the temporal pole while the more posterior left ATL ROI's response reflects sensitivity to sentence-structure. Previous studies of both native sign and spoken languages do find ATL regions sensitive to semantic modulations (Petitto et al., 2000; MacSweeney et al., 2008; Chan et al., 2011; Mayberry et al., 2011; Visser and Lambon Ralph, 2011; Leonard et al., 2013), and these semantic effects in the ATL also are evident in L1 and L2 of spoken language bilinguals (Leonard et al., 2010, 2011). Regardless of the exact sentence-processing resource driving the right ATL's similar response to both L1 English and L2 ASL sentences, it is clear that L1 English sentence-processing resources in the ATL are adapting to the visual-spatial nature of ASL sentence structure.

### Posterior superior temporal lobe

The left posterior superior temporal gyrus (pSTG) is frequently implicated in sentence processing and is known to be sensitive in spoken languages to syntactic structure differences (Humphries et al., 2006; Thothathiri et al., 2012; Griffiths et al., 2013; Wilson et al., 2014) as well as semantic and prosody manipulations (Humphries et al., 2005, 2006). Numerous other studies also implicate portions of the pSTG in syllable-level phonological, semantic, and auditory-motor integration processes (see Hickok and Poeppel, 2007). Thus, it is evident from previous work in spoken languages that the pSTG is a functionally diverse region in regards to its contributions to speech processing. In the present study we found left pSTG regions to more activated by English sentences than word lists and by ASL sentences than word lists (ASL sentences also selectively engaged the right pSTG). The left pSTG ROI more activated by English sentences than words lists demonstrated a main effect for structure (both sentences >both word lists) as well as a main effect of language (English >ASL). Together these findings may reflect that a portion of the pSTG is engaged in sentence-level syntactic processing regardless of proficiency or modality, but with a preference for L1. It also is possible that our findings reflect subvocal rehearsal or translation of the ASL sentences to facilitate comprehension, thereby engaging the left pSTG, in which there are known to be subregions that are involved in phonological processing and verbal working memory (Buchsbaum et al., 2011). It also is possible that the preference for English over ASL in left pSTG may reflect a portion of this region being specific to spoken languages, in that it is most sensitive to phonological processing, coinciding with previous work implicating the pSTG more in orthographically transparent languages (i.e., more grapheme to phoneme conversions) than less transparent language (e.g., Italian vs. English; Paulesu et al., 2000). Future work is needed to explore these possibilities, but altogether our findings support the flexibility of the pSTG's sentence-processing regions to the visual-spatial syntactic structures learned during late L2 ASL, as well as likely the specificity of pSTG phonological processing regions to spoken language processing.

In our exploratory analysis of activations correlated with ASL proficiency, activation in the left posterior superior temporal gyrus to ASL sentences was associated with greater ASL proficiency. Recent neuroimaging studies of spoken L2 learning also implicate the pSTG in L2 proficiency. For example, Chai et al. (2016) resting-state fMRI study of native English-speaking adults learning French found that greater resting-state functional connectivity prior to L2 learning between the left posterior superior temporal gyrus and the anterior insula/frontal operculum (AI/FO, a region adjacent to Broca's area) was associated with eventual greater proficiency in L2 lexical retrieval. Kuhl et al. (2016)'s diffusion tensor imaging study of Spanish-English bilinguals also implicates left superior temporal/inferior parietal regions; they found that higher density and lower diffusivity of white matter pathways underlying these areas were significantly correlated with more years of L2 experience. Thus, it is possible that the pSTG activations associated with increased L2 ASL proficiency reflect greater vocabulary or lexical knowledge.

### Parietal lobe

Previous studies of early bimodal bilinguals and deaf signers have both found parietal regions activated more by sign than spoken language (Newman et al., 2002; Emmorey et al., 2005; Pa et al., 2008). The authors of these studies have suggested that their parietal findings reflect a sign language-specific resource that may be due to modality-specific organization of sensory-motor integration cortex in parietal cortex (Pa et al., 2008). Our present findings coincide with this modality-specific organization of parietal cortex: we found bilateral superior parietal and left inferior parietal / post-central gyrus regions to be more activated by L2 ASL than L1 English sentences, as well as more inferior bilateral parietal regions that show the reverse pattern (L1 English > L2 ASL sentences). Our parietal lobe results suggest that the “ASL-specific” parietal lobe involvement identified in previous studies of early bimodal bilinguals does not seem to be dependent upon age of acquisition. The increased response to ASL sentences than English sentences in the bilateral parietal lobes may be related to the increased spatial hand movements in the ASL sentences to convey grammatical information (subject, object, etc.) (Emmorey et al., 2005), compared to word lists that comparatively lack such movements. Our lack of finding of a right hemisphere parietal involvement for ASL coincides with Newman et al. (2002) findings that this neural correlate of ASL is related to an early age of acquisition.

### Hemispheric laterality of ASL sentence processing

One area of ongoing debate regarding the neurobiology of sign language is the right hemisphere's involvement in sign languages compared to spoken languages. There is evidence to suggest that the right hemisphere is more involved in sign languages than spoken languages, possibly because of the visual-spatial nature of sign languages, for which the right hemisphere is thought to be specialized (Bavelier et al., 1998; Newman et al., 2002; Emmorey et al., 2005). More bilateral activation has been reported during sign language comprehension and production than in spoken language (Emmorey et al., 2014). However, right hemisphere damage resulting in visual-spatial deficits does not necessarily impair sign language abilities of native deaf signers (Hickok et al., 1998a).

We found that L1 English and L2 ASL sentences both activated large bilateral fronto-temporo-parietal networks, as seen in numerous previous studies of spoken language bilingualism, mostly independent of age of acquisition or proficiency (Perani and Abutalebi, 2005; Ge et al., 2015) as well as in both spoken and signed languages in monolinguals (Hickok and Poeppel, 2000, 2004, 2007; MacSweeney et al., 2008). We did not find any evidence of a right hemisphere preference for the L2 ASL vs. L1 sentences, conflicting with some previous studies of both spoken language bilingualism and bimodal bilingualism that have found greater right hemisphere involvement for L2 compared to L1, particularly with later age of acquisition and/or lower L2 proficiency (e.g., in novice L2 learners; Dehaene et al., 1997; Meschyan and Hernandez, 2006) as in our participant sample. One possible reason for this discrepancy is task: our task was a passive viewing/reading task, whereas much of the previous work implicating ASL-specific right hemisphere involvement involved some type of active task (e.g., anomaly detection, sign/word repetition, recognition test) (Newman et al., 2002; Pa et al., 2008; Emmorey et al., 2014), which might lead to increased activation outside of the left-lateralized language network: greater effort during a variety of language tasks has been found to elicit greater right hemisphere recruitment (Just and Varma, 2002).

There was only one right hemisphere activation cluster, in the right middle frontal gyrus adjacent to the right IFG, in which activation in response to ASL sentences was correlated with ASL proficiency. This finding may reflect additional recruitment of cognitive resources being associated with greater ASL proficiency in our cohort of novice ASL learners, as the right IFG and adjacent prefrontal regions are frequently implicated in increased task-related cognitive demands and greater effort during language tasks (Just and Varma, 2002; Goghari and MacDonald, 2009; Prat et al., 2011) and in L2 vs. L1 tasks (Wartenburger et al., 2003; Reiterer et al., 2011). Our mostly null results in the right hemisphere related to proficiency should be interpreted with caution as our study was likely underpowered for correlational analyses. But our null results also may be related to the nature of our ASL stimuli: in deaf signers, Newman et al. (2010b) report greater right hemisphere involvement for ASL sentences with narrative cues (often involving head movements and facial expressions), which our ASL sentences mostly lacked. Another consideration is that stimulus-locked activation measurements may not best reflect right hemisphere contributions to ASL; functional connectivity analyses indicate that signers have greater functional connectivity between right temporal and inferior parietal regions than non-signers (Malaia et al., 2014), this may suggest more efficient involvement of the right hemisphere in signers' processing of ASL that may not be fully reflected in activation differences quantified in our subtraction analyses. It also is possible that in fact the right hemisphere is not engaged in ASL processing even as a function of proficiency because our cohort is still relatively novice learners; this would coincide with previous findings by Malaia et al. (2012) that the right hemisphere is not activated by ASL signs more than non-linguistic gestures in non-signers.

### Future directions

Our ASL sentences contained two types of syntactic manipulations, inflectional morphology (as indicated by spatial relations) and word-order. Future studies are needed to determine if these two properties differentially affect L2 ASL proficiency and the brain regions recruited during ASL sentence comprehension in novice signers. Previous work in deaf native signers suggests that ASL inflectional morphology and word-order grammatical information are supported by two distinct but overlapping networks: Newman et al. (2010b) found that in ASL inflectional morphology particularly engaged bilateral ATL, inferior frontal, and basal ganglia regions (i.e., regions known to be engaged in combinatorial processes and syntactic processing in spoken languages), while ASL sentences with critical word-order grammatical information activated regions often associated with working memory, including regions in the dorsolateral prefrontal cortex (including the inferior frontal gyrus) and inferior parietal lobe. Differentiating between inflectional morphology vs. word order contributions may also provide insight into the contributions of the medial structures including the basal ganglia and hippocampus that in the present study were found to be more engaged by ASL than English stimuli. These subcortical structures' greater involvement in ASL than English may reflect increased memory retrieval, sequencing or combinatorial processes that L2 sentence comprehension requires (Newman et al., 2010a; Leonard et al., 2011), and it is likely that the different grammatical properties recruit these resources differently.

Another potentially meaningful extension of the present study is to conduct a longitudinal study with a similar cohort. The present study collected data post-ASL instruction, thus, it is unknown how our subjects' brains differed prior to ASL exposure. Ample previous work indicates that it is unlikely that the activation patterns we found in response to ASL would exist prior to any ASL exposure, as the subjects would extract very little, if any, semantic or syntactic information from the perceived “gestures” and thus would not fully recruit language networks (Malaia et al., 2012; Leonard et al., 2013; Kovelman et al., 2014). However, there is emerging evidence from spoken L2 learning studies that the integrity of functional and structural brain networks prior to L2 learning are correlated with eventual L2 proficiency (Chai et al., 2016; Kuhl et al., 2016). Thus, a future longitudinal study, with a larger sample size to increase power of correlation analyses, and at least two pre- and post-L2 learning data points, is needed to better characterizes the neural signatures of L2 sentence comprehension and ASL proficiency.

## Conclusion

The present fMRI study examined the functional neuroanatomy of ASL sentence comprehension in adult learners of ASL, particularly how L1 spoken language sentence comprehension networks respond to L2 ASL sentences. We replicate previous work in native signers as well as in spoken language late L2 learners, in that there is a high degree of overlap in the functional neuroanatomy of L1 and L2 sentence comprehension, despite L1 and L2 differences in modality and proficiency. Our results, with previous early bimodal and late spoken language bilingual work providing context, indicate that L1 spoken language sentence-processing regions can adapt to support syntactic structures in a different modality beyond the critical language periods in childhood. We find that within the L1 sentence processing network, Broca's area, the left anterior temporal lobe, and the left posterior superior temporal gyrus each respond differently to L2 ASL sentences. L1 sentence resources in Broca's area were significantly more responsive to L2 sentences, whereas left ATL regions exhibited no significant difference between L1 and L2 sentences, and posterior superior temporal regions exhibited greater preference for the L1 than L2 sentences. We suggest that the engagement of Broca's area to sentence processing may be related to increased cognitive demands associated with the L2 sentences, whereas the responses of L1 sentence regions in the ATL and pSTG to L2 sentences reflect L1 syntactic or combinatorial semantic processes being recruited for L2 sign comprehension. We also found that ASL sentence comprehension proficiency in late L2 learners may be correlated with increased activation in L1 sentence regions and decreased activation in visual-spatial regions in response to ASL sentences, but the nature and origin of these proficiency-related functional differences requires further study.

## Author contributions

LJ, YY, SM, MF, PH, and CR contributed to the design of the study. LJ, YY, SM, MF, LB, and CR were critical in implementing and analyzing the data. All authors contributed to data interpretation and manuscript preparation.

### Conflict of interest statement

The authors declare that the research was conducted in the absence of any commercial or financial relationships that could be construed as a potential conflict of interest.

## Footnotes

^1^It is noteworthy though that sign abilities and visuospatial abilities have been found to dissociate in some native deaf signers following a stroke (Hickok et al., 1998b), but it is unclear if this dissociation would hold in late L2 learners of ASL.

^2^The ATL also is regularly implicated in multi-modal semantic processing (Hodges et al., 1992, 1999; Mummery et al., 2000; Simmons and Martin, 2009), although the activation foci are often anterior to those sensitive to sentence structure (Rogalsky, 2016).
